# Reliability of Transcutaneous Measurement of Renal Function in Various Strains of Conscious Mice

**DOI:** 10.1371/journal.pone.0071519

**Published:** 2013-08-19

**Authors:** Daniel Schock-Kusch, Stefania Geraci, Esther Ermeling, Yury Shulhevich, Carsten Sticht, Juergen Hesser, Dzmitry Stsepankou, Sabine Neudecker, Johannes Pill, Roland Schmitt, Anette Melk

**Affiliations:** 1 Medical Research Centre, Medical Faculty Mannheim, University of Heidelberg, Mannheim, Germany; 2 Department of Nephrology, Hannover Medical School, Hannover, Germany; 3 Department of Kidney, Liver and Metabolic Diseases, Children's Hospital, Hannover Medical School, Hannover, Germany; 4 Experimental Radiation Oncology, Medical Faculty Mannheim, University of Heidelberg, Mannheim, Germany; 5 Mannheim Pharma and Diagnostics GmbH Mannheim; Germany; Mario Negri Institute for Pharmacological Research and Azienda Ospedaliera Ospedali Riuniti di Bergamo, Italy

## Abstract

Measuring renal function in laboratory animals using blood and/or urine sampling is not only labor-intensive but puts also a strain on the animal. Several approaches for fluorescence based transcutaneous measurement of the glomerular filtration rate (GFR) in laboratory animals have been developed. They allow the measurement of GFR based on the elimination kinetics of fluorescent exogenous markers. None of the studies dealt with the reproducibility of the measurements in the same animals. Therefore, the reproducibility of a transcutaneous GFR assessment method was investigated using the fluorescent renal marker FITC-Sinistrin in conscious mice in the present study. We performed two transcutaneous GFR measurements within three days in five groups of mice (Balb/c, C57BL/6, SV129, NMRI at 3–4 months of age, and a group of 24 months old C57BL/6). Data were evaluated regarding day-to-day reproducibility as well as intra- and inter-strain variability of GFR and the impact of age on these parameters. No significant differences between the two subsequent GFR measurements were detected. Fastest elimination for FITC-Sinistrin was detected in Balb/c with significant differences to C57BL/6 and SV129 mice. GFR decreased significantly with age in C57BL/6 mice. Evaluation of GFR in cohorts of young and old C57BL/6 mice from the same supplier showed high consistency of GFR values between groups. Our study shows that the investigated technique is a highly reproducible and reliable method for repeated GFR measurements in conscious mice. This gentle method is easily used even in old mice and can be used to monitor the age-related decline in GFR.

## Introduction

Several approaches for the transcutaneous measurement of glomerular filtration rate (GFR) in laboratory animals have been developed based on fluorescent markers [1]–[13].

All of them allow the measurement of GFR without blood and/or urine sampling and are based on quantifying the elimination kinetics of fluorescent exogenous markers. Generally, these transcutaneous methods were validated against a gold standard plasma clearance procedure. Due to the independence of blood and/or urine samples transcutaneous methods allow repeated GFR measurements in short time intervals in the same animal. Such repeated measurements of GFR are of high interest e.g. in preclinical nephrotoxicity studies of novel medical agents or investigations regarding acute kidney injury (AKI) models and development of AKI treatment regimens. Besides validation against a gold standard the repeatability (self-consistency) of a new method is of utmost relevance, as variations of measured results could be wrongly misinterpreted as e.g. disease progression or treatment outcome. So far, no investigations of repeatability of transcutaneous GFR measurements in the same animal exist. Hence, the repeatability of a transcutaneous method for GFR assessment was evaluated for the first time [14]. The method, allowing measurement in conscious, freely moving mice, is based on the transcutaneous measurement of the elimination kinetics of the exogenous GFR marker FITC-Sinistrin [2]–[4], [7], [8], [10], [14], [15].

Two GFR measurements within three days were performed in five different groups of mice. The collected data were evaluated regarding day-to-day reproducibility of the method as well as inter- and intra-strain variability of GFR and the impact of older age on these parameters [16]. In addition, we assessed GFR again in two additional groups of mice that were purchased at a later time point from the same supplier to test for GFR consistency in the breeding colony and to validate our method over a longer time interval.

## Materials and Methods

### Two transcutaneous GFR measurements were performed in four strains of male mice at 3–4 months of age

Balb/c: n = 15, 29.7±1.2 g body weight (b.w.); C57BL/6: n = 10, 23.9±1.5 g b.w.; SV129: n = 10, 23.6±1.8 g b.w.; NMRI: n = 6, 45.3±1.4 g b.w.; as well as in one group of male mice aged 24 months: C57BL/6: n = 12, 30.9±1.3 g. All mice were purchased from Janvier, France.

In addition, we purchased and investigated additional groups of young (3–4 months, n = 21, 25.9±1.4 g b.w.) and old male C57BL/6 mice (24 months, n = 19, 29.8±3.1 g b.w.). Comparable groups of mice were purchased at same age and from the same breeder (Janvier, France) one year later (C57BL/6: 3–4 months, n = 20, 26.3±0.9 g b.w. and 24 months, n = 23, 30.5±1.9 g b.w.). In all these groups GFR measurements were performed once.

The protocol was approved by the Committee on the Ethics of Regierungspräsidium Karlsruhe (Permit Number: 35-9185.81/G19/11).

### Transcutaneous bolus clearance

The transcutaneous measurement was performed as previously described [14]. In brief, the miniaturized fluorescence detector (NIC-Kidney; Mannheim Pharma & Diagnostics GmbH, Mannheim, Germany) was fixed on a depilated region on the back of the mice using a double-sided adhesive patch (Lohmann GmbH & Co. KG, Neuwied, Germany). The preliminary depilation and the FITC-Sinistrin injection were performed under short isoflurane (Abbott Laboratories, Abbott Park, USA) anesthesia.

The measurement started with activation of the device before FITC-Sinistrin (7.5 mg/100 g b.w. dissolved in 0.25 mL NaCl 0,9%; Mannheim Pharma & Diagnostics GmbH, Mannheim, Germany) was injected, in order to measure the background signal for 1 min. Starting from the marker injection, the data acquisition lasted 60 min.

### GFR calculation

Transcutaneous GFR was calculated using the half-life (t_1/2_) derived from the rate constant of the single exponential elimination phase of the fluorescence-time curve and a semi-empirical mouse-specific conversion factor established previously [14].
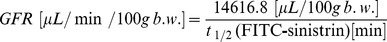
(1)


### Statistical analysis

For day-to-day reproducibility, i.e. intra-strain repeatability, Bland-Altman plots were generated and 97.5% limits of agreement were calculated from mean –2× SD (standard deviation) and mean +2× SD [17]: As further measures of agreement of day-to-day reproducibility, we calculated the coefficient of variation (CV, calculated as SD/M*100%). Multi-factor ANOVA was used to check for influences of the factors day, and the interaction between day and strain.

## Results

### Day-to-day reproducibility (intra-strain repeatability)

**Figure 1:**
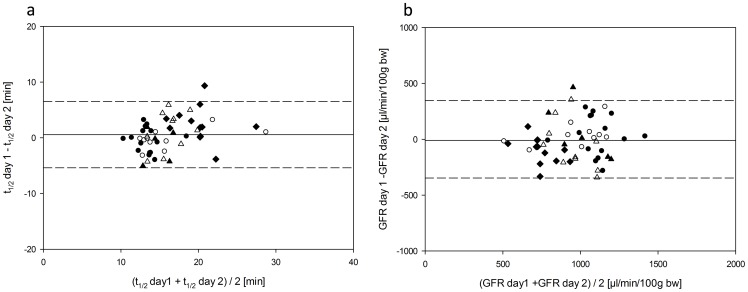
Bland-Altman plots of individual half-life (t_1/2_) (a) and GFR (b) after FITC-Sinistrin injection in five groups of mice (three to four months of age: Balb/c: filled circles, C57BL/6: open circles, SV129: open triangles, NMRI: open diamonds C57BL/6 24 months of age: filled diamonds).

Across all experimental groups for FITC-Sinistrin excretion, mean half-life (t_1/2_) ± SD was 16.4±4.5 and 15.9±3.6 min as well as mean GFR ± SD was 951±235 and 961±187 µL/min/100 g b.w. at the first and second day of measurement, respectively. Mean difference between repeated measurements was 0.56±3 min and −10.5±178 µL/min/100 g b.w., respectively. The CV between measurements was 10.88% for t_1/2_ and 11% for GFR. The 97.5% limit of agreement (LoA) was −5,4 to 6.52 min and −368 to 347 µL/min/100 g b.w. (Figure 1a and b). The data on day-to-day reproducibility for the different groups investigated (Table 1) indicate a good agreement of the repeated measurements.

**Table 1:** Two consecutive assessments of FITC-Sinistrin half-life (t_1/2_) and GFR as well as its coefficient of variation (CV) and Bland Altman parameter in five groups of male mice using transcutaneous measurement.

group	body weight (g)	t1/2 [min]		CV [%]	Bland-Altman parameter	
		day1	day2		bias	97.5% LoA
Balb/c (n = 15)	29.7±1.2	13.2±2.0	13.7±2.2	9.5	−0.45	−4.85 to 3.94
C57Bl/6 (n = 10)	23.9±1.5	16.1±5.7	16.4±4.8	5.8	−0.28	−3.88 to 3.33
C57Bl/6 24m (n = 12)	30.9±1.3	20.3±3.2	18.8±3.6	11.2	2.59	−3.89 to 9.07
SV129 (n = 10)	23.5±1.8	17.1±3.3	15.7±2.2	14.2	1.40	−5.78 to 8.59
NMRI (n = 6)	45.3±1.4	14.1±2.4	14.9±2.4	12.1	−0.75	−7.02 to 5.52

group	body weight (g)	GFR [µL/min/100g bw]		CV [%]	Bland-Altman parameter	
		day1	day2		bias	97.5% LoA
Balb/c (n = 15)	29.7±1.2	1127.4±159.6	1093.4±169.1	9.3	33.94	−323.80 to 391.68
C57Bl/6 (n = 10)	23.9±1.5	986.4±248.8	944.1±198.3	5.6	42.30	−179.98 to 264.57
C57Bl/6 24m (n = 12)	30.9±1.3	698.8±95.0	801.9±143.0	13.0	−103.2	−342.0 to135.80
SV129 (n = 10)	23.5±1.8	889.1±196.8	948.1±128.9	15.2	−59.01	−505.7 to 387.69
NMRI (n = 6)	45.3±1.4	1059.7±188.5	1004.5±158.6	11.3	55.18	−446.25 to 556.62

Values given as mean ± standard deviation, LoA: limits of agreement.

### Intra-group variability of FITC-Sinistrin excretion

#### t_1/2_ and GFR.

**Figure 2:**
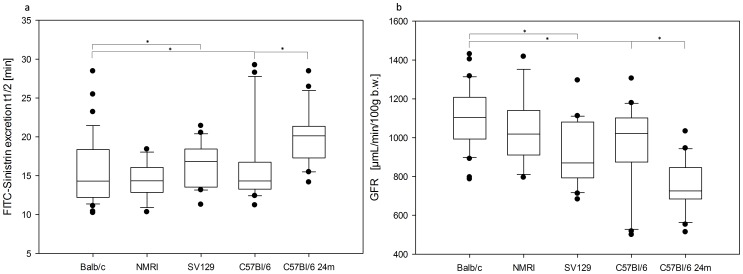
Intra-strain variability of half-life (t_1/2_) and GFR after i.v. injection of FITC-Sinistrin assessed in two consecutive measurements per mail mouse (three to four months of age: Balb/c: n = 15, NMRI: n = 6, SV129: n = 10, C57BL/6: n = 10; C57BL/6 24 month (m) of age: n = 12,). The boundary of the box closest to zero indicates the 25th percentile, a line within the box marks the median, and the boundary of the box farthest from zero indicates the 75th percentile. Whiskers (error bars) above and below the box indicate the 90th and 10th percentiles. In addition, outliers are graphed by dots. (*: p<0.05 by two tailed t-test).

To investigate intra-group variability of FITC-Sinistrin excretion, t_1/2_ and GFR are depicted in Figure 2.

### Comparison between groups

Multi-factor ANOVA revealed that the day-related factors (day and day×group) have no significant effect on the FITC-Sinistrin t_1/2_ (p-value day×group  = 0.72; p-value day = 0.77). However, there is a significant influence of the group factor (p-value group <0.0001).

Comparison of the accumulated data of all examined groups with each other using a two tailed Student's t-test is summarized in Figure 2.

### GFR consistency in animals with similar age from the same supplier at different dates

**Figure 3:**
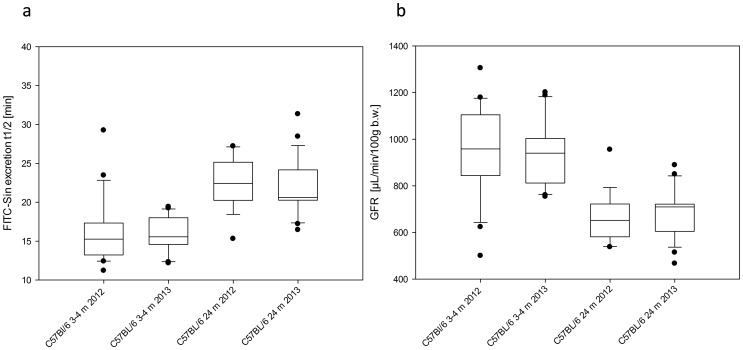
Consistency of FITC-Sinistrin excretion t_1/2_ (a) and GFR (b) in mice from the same supplier at distant time points. Transcutaneous measurements were performed in male young (n = 20) as well as in a 24 months (m) old C57BL/6 (n = 18) mice in 2012. One year later (2013) comparable groups of C57BL/6 mice (young: n = 23; old: n = 24) from the same breeder (Janvier, France) were investigated in the same way. No significant difference between the two measurement dates in the respective groups were found by two tailed t-test (3–4 m: p = 0.82, 24m: p = 0.53) for both parameters. The boundary of the box closest to zero indicates the 25th percentile, the line within the box marks the median, and the boundary of the box farthest from zero indicates the 75th percentile. Whiskers (error bars) above and below the box indicate the 90th and 10th percentiles. In addition, outliers are graphed by dots.

In a yearly difference two groups of mice (young and old C57BL/6) were purchased the same supplier. GFR measurements revealed similar results with no significant differences between the respective groups using a two tailed Student's t-test (Figure 3).

## Discussion

Measurement of GFR is of critical importance for detecting kidney injury, predicting outcome, adapting drug dosages, and monitor therapeutic management in clinical setting. In the experimental setting GFR is also important for e.g. phenotyping of animal models, testing treatment strategies or analyzing nephrotoxicity. Currently, plasma creatinine concentration is often used to estimate GFR in mouse models. However, plasma creatinine has been shown to be a poor marker of GFR in mice [18].

To monitor changes of renal function especially in the context of AKI and nephrotoxicity it is instrumental to assess GFR repeatedly in short time intervals. Classical plasma sampling based methods or novel techniques like the intravenous applied fluorescent probe described by Molitoris et. al. are not feasible in conscious mice in this respect [19]. The data in this report demonstrate that the method used for transcutaneous GFR measurement meets the requirements for frequent and repeated GFR assessment in mice [14].

Using t_1/2_ of FITC-Sinistrin as the key basic read-out, the missing of statistically significant differences in day-to-day measurements in all groups indicate a high consistency. A high reproducibility of our method was also reflected by the Bland-Altman analysis showing almost no bias between the two measurements. Moreover, the mean CV of 10.8% for t_1/2_ and 11% for GFR, respectively, as a measure of reproducibility is in the range known from repeated GFR measurements in humans assessed by a variety of other techniques [20]–[24].

As the conversion of t1/2 to GFR is formula based the comparison between t1/2 and GFR are nearly identical, as expected.

As one would assume, significantly slower excretion t_1\2_ is detected in the 24 month old C57BL/6 compared to the 3–4 month old group in both experiments. This reduction of GFR with older age is in line with observations by Hackbart et. al [25]. In contrast to previous data, we found a rather tight intra-group variability. Old fragile mice do profit from our gentle method that puts far less strain on the animal than maintenance over 24 hours in a metabolic cage. An important additional finding is the consistency of the GFR measurements in mice of the same age that come from the same supplier at different time points. This holds true even for older animals where age-related differences in GFR-decline could have been possible.

It is important to note that the magnitude of CV and 97.5% LoA of the Bland-Altman analysis do not only result from measurement errors, e.g. due to mechanical pressure applied to the device [14], but also from known day-to-day variability of GFR. Day-to-day variability is most pronounced in healthy animals with normal renal reserve capacity and caused by adaption of kidneys to issues like food intake (protein), hydration status or blood pressure or renal blood flow [26]. Therefore, the observed differences in CV between the strains can be explained at least in parts by inter-strain differences in the renal reserve capacity. To minimize the impact of the circadian rhythm of GFR the measurements in the animals were performed at the same time of day.

A drawback of the technique is that GFR is not calculated directly from the assessed data, but an empirically derived conversion formula is needed. This formula is based on the assumption that the extra cellular volume (ECV)/100 g b.w. is comparable in all investigated groups. Even a change in ECV as long as it affects the whole organism does not influence the final excretion t_1/_ because only the distribution time of the marker (time to max concentration, time to reach single exponential decay phase) and the overall measured signal intensity are affected. However, in case there is a different ECV due to e.g. local edema which is filled and cleared with different rates to other ECV compartments, this may potentially influence the measurements. The later is also true for the classical plasma sampling based bolus clearance experiments, because these models do not consider this extra compartment. The only precise way of measuring GFR thereby is by the use of only constant infusion techniques. We consider the short gas anaesthesia required for device mounting and substance injection as a negligible problem as it takes about 15 min until the single exponential decay phase is reached.

In summary, the results of this study clearly indicate that the investigated transcutaneous technique for GFR assessment is a reproducible and reliable method for repeated GFR measurements. In contrast to other transcutaneous methods, the measurements can be performed in conscious, freely moving mice excluding the high impact of anesthesia on GFR. This is especially important when dealing with old, mostly fragile mice. Blood or urine samples are not required, which makes it an appropriate method for multiple measurements in the same animal in short time intervals.
